# Fuzzy Entropy Analysis of the Electroencephalogram in Patients with Alzheimer’s Disease: Is the Method Superior to Sample Entropy?

**DOI:** 10.3390/e20010021

**Published:** 2018-01-03

**Authors:** Samantha Simons, Pedro Espino, Daniel Abásolo

**Affiliations:** 1 Centre for Biomedical Engineering, Department of Mechanical Engineering Sciences, Faculty of Engineering and Physical Sciences, University of Surrey, Guildford GU2 7XH, UK; 2 Hospital Clínico Universitario de Valladolid, 47003 Valladolid, Spain

**Keywords:** Alzheimer’s disease, electroencephalogram, non-linear analysis, complexity, irregularity, Fuzzy Entropy, Sample Entropy

## Abstract

Alzheimer’s disease (AD) is the most prevalent form of dementia in the world, which is characterised by the loss of neurones and the build-up of plaques in the brain, causing progressive symptoms of memory loss and confusion. Although definite diagnosis is only possible by necropsy, differential diagnosis with other types of dementia is still needed. An electroencephalogram (EEG) is a cheap, portable, non-invasive method to record brain signals. Previous studies with non-linear signal processing methods have shown changes in the EEG due to AD, which is characterised reduced complexity and increased regularity. EEGs from 11 AD patients and 11 age-matched control subjects were analysed with Fuzzy Entropy (FuzzyEn), a non-linear method that was introduced as an improvement over the frequently used Approximate Entropy (ApEn) and Sample Entropy (SampEn) algorithms. AD patients had significantly lower FuzzyEn values than control subjects (*p* < 0.01) at electrodes T6, P3, P4, O1, and O2. Furthermore, when diagnostic accuracy was calculated using Receiver Operating Characteristic (ROC) curves, FuzzyEn outperformed both ApEn and SampEn, reaching a maximum accuracy of 86.36%. These results suggest that FuzzyEn could increase the insight into brain dysfunction in AD, providing potentially useful diagnostic information. However, results depend heavily on the input parameters that are used to compute FuzzyEn.

## 1. Introduction

Alzheimer’s disease (AD) is a form of dementia that is characterised by progressive impairments in cognition and memory [1]. The cause of AD is not known [2], and the course of the disease can last several years before death [1]. As AD is currently the most prevalent dementia worldwide [3,4] the impact of the disease is significant. Current clinical diagnosis is based on the National Institute of Neurological and Communicative Disorders and Stroke and Alzheimer's Disease and Related Disorders Association (NINCDS-ADRDA) criteria [5], and, although definite diagnosis is only possible by necropsy, a differential diagnosis with other types of dementia would be of great use.

The electroencephalogram (EEG), a recording of the electrical activity of the brain, shows great potential to characterise changes in brain activity as a result of AD. There are several reasons for this; the first being that AD is a cortical dementia [1] and, therefore, changes to the electrical activity of the brain resulting from AD could be registered on EEGs. Furthermore, the EEG can be recorded non-invasively, with portable equipment and at much lower cost than other imaging techniques that are used in AD diagnosis. Therefore, the application of signal processing algorithms to extract features from EEG signals may help in the characterisation of the changes that are associated with AD. In fact, several EEG features appear to be abnormal in AD patients, where a shift of the power spectrum to lower frequencies, a decrease of coherence among cortical areas, perturbed synchrony, and reduced complexity have been observed (for detailed reviews, please see [1,6]), although in the early stages of the disease, the EEG may show similar features to that of age-matched healthy controls [7]. In spite of these findings, there is room for the introduction of novel signal processing techniques for further study of the EEG. In particular, entropy algorithms quantifying irregularity in data could be useful to capture subtle changes in the EEG that might be caused by AD.

Different entropy algorithms have been introduced over the years to characterise the EEG, with greater entropy being associated with increased irregularity in the EEG. Embedding entropies are algorithms where entropy is used to provide information about how the EEG signal fluctuates with time by comparing the time series with a delayed version of itself [8]. The introduction of Approximate Entropy (ApEn) by Pincus [9] made the reliable characterisation of the entropy of short and noisy biomedical signals possible in ways that were, up until its introduction, not achievable. ApEn measures the regularity in data by examining time series for similar epochs and assigning a non-negative number to the sequence, with larger values corresponding to more complexity or irregularity in the data [9]. Given a time series with *N* samples, a sample length *m* and a tolerance window *r*, ApEn(*m*, *r*, *N*) measures the logarithmic likelihood that samples of patterns that are close (within *r*) for *m* contiguous observations remain close (within the same tolerance width *r*) on subsequent incremental comparisons [9]. The ApEn algorithm counts each sequence as matching itself to avoid the occurrence of *ln*(0) in the calculations. The effect of self-matches provides a biased estimate of entropy, giving a false impression of determinism [10]. Furthermore, ApEn values depend heavily on the record length, with ApEn being lower than expected for short time series, and it also lacks consistency when different input parameter values are used to evaluate the same time series [11]. It was subsequently superseded by Sample Entropy (SampEn), as introduced by Richman and Moorman [11]. As it is also the case with ApEn, two input parameters, *m* and *r*, must be specified to compute SampEn. SampEn(*m*, *r*, *N*) is the negative logarithm of the conditional probability that two sequences similar for *m* point vectors remain similar at the next point, where self-matches are not included in calculating the probability. SampEn would be lower for signals that show a higher degree of self-similarity, i.e., more regular. In addition to overcoming some of the limitations of ApEn, SampEn is easier to compute [11]. For both ApEn and SampEn, the recommended range of values for input parameters are *m* = 1 or 2 and *r* between 0.1 and 0.25 times the standard deviation of the input data [11].

Different algorithms that are attempting to improve SampEn have been suggested. Quadratic Sample Entropy (QSE) was introduced to reduce the influence of the arbitrary constants *m* and *r* on SampEn and to reduce the skewing of results when either the top or the bottom of the conditional probabilities was very small or very large [12]. Input variables are the same (i.e., a sample length *m* and a tolerance window *r*) though the recommended values are different, with *r* not being limited to the range suggested for ApEn and SampEn. Another attempt to improve SampEn was with the introduction of Fuzzy Entropy (FuzzyEn) [13], based on the concept of fuzzy sets to determine a fuzzy measurement of similarity of two vectors based on their shapes.

In this pilot study, FuzzyEn was used to characterise the broadband activity in the EEG of patients with AD. It was hypothesised that FuzzyEn would identify differences between the entropy of EEG signals from AD patients and age-matched control subjects, and that these differences could be used to help in the classification of EEG signals with respect to their class (AD patient or control subject). The quality of the classification would be evaluated using receiver operating characteristic (ROC) curves [14]. Furthermore, FuzzyEn results would be compared with SampEn to ascertain whether the claims that the former is a superior method to the latter hold true in the context of EEG analysis in AD.

The outline of the paper is as follows. Section 2 describes the EEG database that is used in this study and introduces FuzzyEn. Results obtained with all of the input parameters tested in this pilot study are presented in Section 3, whilst the discussion of the findings, focusing on a comparison of the results obtained with FuzzyEn and other related entropies, and the conclusions from this study follow in Section 4.

## 2. Materials and Methods

### 2.1. Subjects and EEG Recording

The database used in this study consisted of 11 patients with a diagnosis of AD (five men; six women; age: 72.5 ± 8.3 years, mean ± standard deviation (SD)), recruited from the Alzheimer’s Patients’ Relatives Association of Valladolid (AFAVA), Spain, and 11 age-matched controls (seven men; four women; age: 72.8 ± 6.1 years, mean ± SD). AD diagnosis was supported by clinical evaluation (clinical history, physical, and neurological examination) and Mini-Mental State Examination (MMSE), which is generally accepted as an effective way to evaluate cognitive function [15], was also performed. The average MMSE score for the AD patients was 13.1 ± 5.9 (mean ± SD), indicating moderate to severe dementia, and the score was 30 for all of the control subjects, indicating no mental impairment. All of the subjects and caregivers gave their informed consent for participation in the study.

EEG signals were recorded with the subjects in a relaxed state with eyes closed at the Hospital Clínico Universitario de Valladolid (Spain) using Oxford Instruments Profile Study Room 2.3.411 EEG equipment and the international 10–20 system with electrodes referenced to the linked ear lobes of each subject. More than 5 min of EEG data were recorded for each subject with a sampling rate of 256 Hz. Two AD patients were taking lorapezam at the time of recording the EEG, but no prominent rapid rhythms were observed in the visual examination of their EEGs. None of the other subjects who took part in the study were using medication that could be expected to influence the EEG.

Artefact-free sections of the EEG signals (split into 5-second epochs with no movement artefacts and no electroencephalographic signs of sleep) were selected by Dr Pedro Espino, the specialist physician that was overseeing the recording of the EEGs. On average, 30.0 ± 12.5 epochs (mean ± SD) were selected from each electrode for each subject. All of the epochs selected were filtered using a Hamming window FIR filter with order 426 and cut-off frequencies at 0.5 Hz and 40 Hz to remove residual noise (DC offset and mains hum) prior to the computation of FuzzyEn. Zero-phase filtering was used to make sure the use of a filter of such high order did not result in edge effects.

### 2.2. Fuzzy Entropy

Both ApEn and SampEn measure the similarity of the vectors being compared using a Heaviside function, which can be represented as:

(1) $$\theta \left(z\right)=\{\begin{array}{c} 1,\;\;if\;z\geq 0\\ 0,\;\;if\;z<0 \end{array}$$

This leads to a two-state binary classifier, where the vectors are either close or not. However, this might not be able to capture in the most appropriate way the boundaries between different classes, which in real biomedical data might be more ambiguous [13]. Therefore, FuzzyEn was introduced to overcome this limitation with a fuzzy function instead of the Heaviside function used to calculate the similarity degree between vectors. 

Since its introduction, FuzzyEn has been used to characterise different types of biomedical signals, such as electromyograms [13,16,17,18], EEGs [19,20], gait [20], or heart rate variability [20,21]. Comparative studies with ApEn and SampEn suggest that FuzzyEn outperforms them [17,19]. Furthermore, recent evidence suggests that FuzzyEn is a robust entropy estimator when there are missing samples in the biomedical signals being analysed [20].

Given *N* data points from a time series {*x*(*n*)} = *x*(1), *x*(2), …, *x*(*N*), FuzzyEn can be calculated using the following algorithm [13]:

1. For 1 ≤ *i* ≤ *N − m +* 1, form *m*-vectors ***X****_m_*(1) … ***X****_m_*(*N − m +* 1) defined as:
  (2) $$X_{m}\left(i\right)=\;\left[x\left(i\right),\;x\left(i+1\right),\;\ldots ,x\left(i+m-1\right)\right]-x0\left(i\right)$$
  These vectors represent *m* consecutive *x* values, commencing with the *i*th point, with the baseline ($x0\left(i\right)=\frac{1}{m}\sum_{j=0}^{m-1}x\left(i+j\right)$) removed.
2. Define the distance between vectors ***X****_m_*(*i*) and ***X****_m_*(*j*), $d_{ij,m}$, as the maximum absolute difference between their scalar components.
3. Given *n* and *r*, calculate the similarity degree $D_{ij,m}$ of the vectors ***X****_m_*(*i*) and ***X****_m_*(*j*) with a fuzzy function:
  (3) Dij,m=μ(dij,m,r)=exp(−(dij,m)nr)
4. Define the function $\phi_{m}$ as:
  (4) $$\phi_{m}\left(n,r\right)=\frac{1}{N-m}\overset{N-m}{\underset{i=1}{\sum }}\left(\frac{1}{N-m-1}\overset{N-m}{\underset{j=1,j\neq i}{\sum }}D_{ij,m}\right)$$
5. We increase the dimension to *m* + 1, form vectors ***X****_m_*_+1_(*i*), and, subsequently, obtain the function $\phi_{m+1}$ repeating steps 2 to 4.
6. For time series with a finite number of samples *N*, FuzzyEn can be estimated with the following equation [13]:
  (5) $$\mathrm{FuzzyEn}\left(m,n,r,N\right)=\ln\phi_{m}\left(n,r\right)-\ln\phi_{m+1}\left(n,r\right)$$

Given that FuzzyEn is based on the original SampEn algorithm, as introduced by Richman and Moorman in [11], it can be therefore computed as the negative logarithm of the conditional probability that two sequences similar for *m* points—where similarity is measured using the fuzzy function introduced in Equation (3), instead of the Heaviside function used in the ApEn and SampEn algorithms—remain similar when the size of the vectors being considered is increased by one. The algorithm, as is also the case with SampEn, does not include self-matches when calculating the probability aforementioned. Thus, it does not show the bias that is associated with ApEn [11]. Furthermore, lower values of FuzzyEn indicate more self-similarity in the time series being characterised with this algorithm.

It is obvious that FuzzyEn values would depend on the values of the input parameters *m*, *n*, *r*, and *N*, and comparisons should only be attempted for fixed values of these parameters. *N* is the length of the time series and is determined, in this particular study, by the sampling frequency of 256 Hz and the epoch length of 5 s. Parameter *m* determines the length of the sequences to be compared, as in ApEn and SampEn. On the other hand, *r* and *n* determine the width and gradient of the fuzzy exponential function.

In principle, larger values of *m* allow for a better reconstruction of the dynamics of the system being characterised. However, the accuracy and confidence of the entropy estimate improve with a greater number of matches of vectors of length *m* and *m* + 1. Therefore, it is usually recommended to choose small values of *m* [11]. 

**Figure 1:**
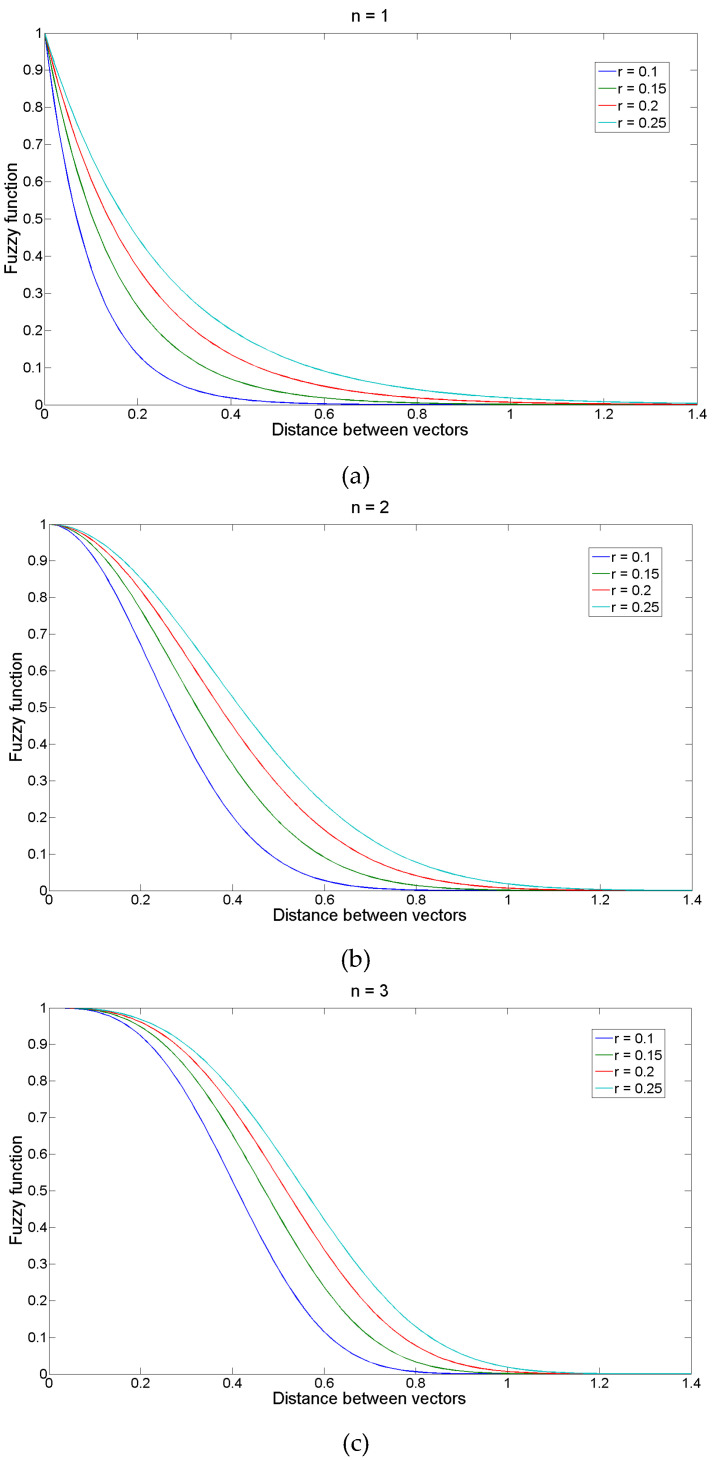
Fuzzy function for *n* = 1 (**a**), *n* = 2 (**b**), and *n* = 3 (**c**) and the different values of *r* used in the study.

Figure 1 shows the changes in the shape of the fuzzy exponential function changes with *n* and *r*. It has been recommended to use small integer values of *n* [13] and set the tolerance width as *r* times the standard deviation (SD) of the original data sequence [11]; the latter would give FuzzyEn scale invariance [9].

Based on these recommendations, in this pilot study, values of *m* = 1 and *m* = 2, *n* = 1, *n* = 2, and *n* = 3, and *r* = 0.1, *r* = 0.15, *r* = 0.2, and *r* = 0.25 times the SD of the original time series were used. This led to 24 variable combinations tested. FuzzyEn was therefore computed using 24 input parameter combinations for channels Fp1, Fp2, F3, F4, C3, C4, P3, P4, O1, O2, F7, F8, T3, T4, T5, and T6.

### 2.3. Statistical Analysis

The distribution of the FuzzyEn results was evaluated with the Lilliefors test. Depending on the results from the Lilliefors test, Student’s *t*-test, or Kruskal-Wallis tests were used to evaluate the statistical significance of differences between groups of subjects at each electrode. In all of the above statistical analyses, statistical significance was at *p* < 0.01.

Results from the electrodes where statistically significant differences between AD patients and controls were found were then analysed with ROC curves, and sensitivity (true positive rate, i.e., percentage of AD patients correctly classified), specificity (true negative rate, i.e., proportion of control subjects correctly identified), accuracy (percentage of total subjects classified precisely), area under the curve, and the optimum threshold (FuzzyEn value that maximises diagnostic accuracy) were computed.

## 3. Results

FuzzyEn was computed for all the 24 input parameter values. Results were averaged based on all of the artefact-free five second epochs within the five-minute period of EEG recordings for the 22 subjects. For all the possible combinations of *m*, *n*, and *r* values, and most electrodes, FuzzyEn was higher for the EEG of control subjects than that for AD patients. The tables in the Supplementary Materials section contain all of the results for the 24 combinations of input parameter values tested.

The results depended heavily on the choice of input parameters. For *n* = 1, the FuzzyEn values were found to follow a normal distribution. Therefore, Student’s *t* Test was used to evaluate the statistical significance of the findings. For all the values of *r* and *m* = 1, FuzzyEn was significantly lower (*p* < 0.01) for AD patients at electrodes Fp1, T6, P3, and O2. With *m* = 2 and all the values of *r*, FuzzyEn was significantly lower (*p* < 0.01) for AD patients at electrodes T6, P3, P4, O1, and O2. These results suggest that AD is associated with a significant decrease of entropy—as estimated by FuzzyEn—in some, but not all, areas of the brain.

For *n* = 2 and *n* = 3 the results did not to follow a normal distribution and the Kruskal-Wallis test was used to evaluate the statistical significance of the findings. With *n* = 2, the number of electrodes where significant differences (*p* < 0.01) between both groups were found dropped significantly when compared to results obtained with *n* = 1. FuzzyEn was significantly lower in AD patients’ EEGs at P3 (with *m* = 1 and all values of *r*, and *m* = 2 and *r* = 0.15, 0.2, and 0.25) and O2 (with *m* = 1 and *r* = 0.1, 0.15, and 0.2, and *m* = 2 and *r* = 0.2, and 0.25). With *n* = 3 and *m* = 1, FuzzyEn was only significantly lower (*p* < 0.01) in AD patients’ EEGs at electrode O2 (for all combinations of *r*). With *n* = 3 and *m* = 2, the only electrode where FuzzyEn was significantly lower (*p* < 0.01) in AD patients was P3, and this only for *r* = 0.2 and *r* = 0.25. Furthermore, the dispersion of FuzzyEn values increased significantly for *n* = 3, suggesting a less reliable entropy estimate (see results in the Supplementary Materials section).

**Figure 2:**
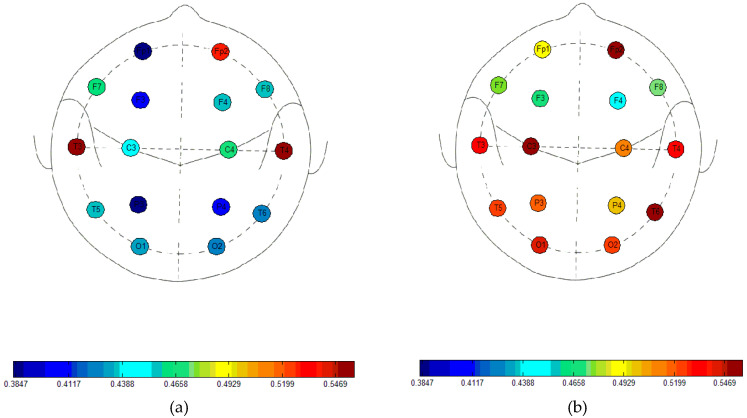
Average Fuzzy Entropy (FuzzyEn) values with *n* = 1, *m* = 2 and *r* = 0.25 times the Standard Deviation (SD) of the time series for Alzheimer’s disease (AD) patients (**a**) and controls (**b**).

Figure 2 summarises the average FuzzyEn values for *n* = 1, *m* = 2, and *r* = 0.25 times the SD of the EEG time series, the combination of input parameters that highlights the biggest differences between both groups for all possible input parameter combinations. The decrease in entropy in AD patients is particularly evident for electrodes that are placed over the parietal, occipital, and temporal regions.

The possible usefulness of FuzzyEn in a diagnostic context was evaluated with ROC curves. The greatest accuracy, at 86.36%, was found when *n* = 1. This was the case in 9 of the 36 electrode and variable combinations where significant differences between the controls subjects and the AD patients had been found using that value of *n*. The largest area under the curve (0.9091) was found at electrode P3 when *n* = 1, *m* = 2, and *r* = 0.1 times the SD of the time series, closely followed by the area at P3, when *n* = 1, *m* = 2, and *r* = 0.25 times the SD of the time series with 0.9008. These did not correspond to the highest accuracy found, with 81.82% accuracy in both cases. Maximum sensitivity was 90.91%, whilst there were some combinations of electrode and input parameter values resulting in 100% specificity. The complete results for FuzzyEn with *n* = 1 are summarised in Table 1.

ROC results when *n* = 2 are summarised in Table 2. For this particular value of the fuzzy function, there were 12 combinations of electrode and input parameter values that showed a significant decrease of FuzzyEn in AD patients. The greatest accuracy was 81.82% and the largest area under the curve was 0.8843. Neither sensitivity nor specificity reached values that were greater than 81.82% in any case.

With *n* = 3, there were even fewer combinations (six in total) of electrode and input parameter values showing a significant decrease of FuzzyEn in AD. Accuracy reached a maximum value of 81.82%, whilst the largest area under the curve was 0.8678 (for an accuracy of 77.27%). As was also the case for *n* = 2, neither sensitivity, nor specificity, exceeded 81.82%. ROC results for these results are held in Table 3.

## 4. Discussion and Conclusions

Resting state EEG activity of 11 AD patients and 11 control subjects was characterised with FuzzyEn in this pilot study. FuzzyEn was introduced to overcome some limitations of ApEn and SampEn [13], in particular, the fact that both algorithms use a Heaviside function to measure the similarity of the embedding vectors from the time series being compared [13].

FuzzyEn was lower in the EEG of AD patients for all possible combinations of *m*, *n*, and *r* values and for most electrodes. The greatest number of electrodes (Fp1, T6, P3, P4, O1, O2) showing significant FuzzyEn differences between the EEG of AD patients and controls were seen when *n* = 1, i.e., the steepest gradient of the exponential function. Furthermore, the highest values of accuracy and area under the ROC curve were also obtained with this value of *n*. Our results suggest that brains affected by AD show a more regular electrophysiological behaviour in the parietal and occipital regions, something that is in agreement with previous studies [22,23,24,25,26].

Relevant findings in the changes in the EEG with AD using this same database include a significant reduction in complexity, as measured with the Lempel-Ziv algorithm, at electrodes T5, P3, P4, and O1, with classification accuracies between 72.73% and 81.82% [22]. Some of those electrodes showing reduced complexity (measured with Lempel-Ziv complexity) coincide with those where a significant decrease of irregularity in AD patients’ EEGs has been highlighted by FuzzyEn. However, it is worth noting that FuzzyEn was able to find differences in a greater number of electrodes than Lempel-Ziv complexity. A significant loss of complexity in the EEG of AD patients at T5, T6, P3, P4, O1, and O2 was also found with a method based on auto-mutual information, with classification accuracies ranging from 81.82% to 90.91% [23], as well as with multiscale entropy (MSE), for which significant differences between the MSE of AD patients and controls were found at F3, F7, Fp1, Fp2, T5, T6, P3, P4, O1, and O2, with accuracies from 77.27% to 90.91% [24].

More relevant though are previous studies with this same EEG database using ApEn [23], SampEn [25], and QSE [26]. It was found that ApEn was significantly lower in AD patients than in controls at electrodes P3, P4, O1, and O2. However, classification accuracies that were obtained using ROC curves at these electrodes ranged from 72.72% to 77.27% (with the latter value found at P3, O1, and O2). The largest area under the curve was 0.8595 (at P3 and O1). Nevertheless, ApEn results should be interpreted with great care, as this is a biased entropy estimator, and, therefore, not as reliable as other algorithms [10,11]. SampEn values were also significantly lower for AD patients’ EEGs than for age-matched controls’ EEGs at O1, O2, P3, and P4. Moreover, with SampEn the classification accuracy obtained with ROC curves reached 77.27% at all of those electrodes, supporting the superior discriminating power of SampEn when compared to ApEn, which could arise from the fact of SampEn being an improvement over ApEn. Nevertheless, the largest area under the curve (0.8595 at O1) was similar to the one that is found with ApEn. These results are also supported by recent findings with QSE, with accuracies of 77.27% at P3, O1, and O2, for *m* = 1, *m* = 2, and a range of values of *r* [26]. All of these results support that EEG activity of AD patients is significantly more regular (less complex) than in a normal brain in the parietal and occipital regions. Table 4 summarises the results obtained with all these methods.

It is worth noting that the detection of a significant decrease of entropy in the EEG of AD patients is heavily dependent on the input parameters that are used in the different entropy estimators. In the case of SampEn, the combination of input parameters that yielded the best results were *m* = 1 and *r* = 0.25 times the standard deviation of the time series [25], whilst for QSE, similar results were obtained with *m* = 1, *m* = 2, and a wide range of values of *r* [26]. On the other hand, for FuzzyEn, the combination of input parameters that resulted in the greatest number of electrodes showing a statistically significant decrease of entropy in AD (five in total: T6, P3, P4, O1, O2) and the highest accuracies, was *n* = 1, *m* = 2, and *r* = 0.25 times the standard deviation of the time series. In fact, with *n* = 1, *m* = 1, and *r* = 0.25, FuzzyEn was significantly lower for AD patients at electrodes Fp1, T6, P3, and O2, but no significant differences were found at P4 and O1, unlike with SampEn or QSE. Therefore, recent claims of FuzzyEn being superior to ApEn and SampEn in the discrimination of EEG signals in AD patients [19] need to be evaluated with great care, as that might not necessarily be the case for all the combinations of input parameters. Furthermore, our results suggest that FuzzyEn becomes a much less reliable entropy estimator when *n* = 3 than when *n* = 1 or *n* = 2.

Our study is not the first time that the concept of FuzzyEn has been used to evaluate the complexity changes in the EEG in AD patients. Fuzzy versions of ApEn and SampEn (the latter corresponding to the algorithm used herein) were used to compute the complexity of the EEG in the delta, theta, alpha, and beta bands [19]. It was shown that the fuzzy entropies could distinguish EEGs of AD patients from those of controls in a better way than ApEn and SampEn, with a significant decrease in the alpha band, particularly at electrodes T3 and T4. A classification accuracy of 88.1% using fuzzy SampEn and a support vector machine classifier was reported. However, results cannot be compared directly to those that are presented above, as the analysis in [19] focused on different EEG frequency bands, with significant differences being found only in the alpha band. In our study, we have characterised the entropy of a much broader bandwidth of the EEG at rest, therefore limiting the impact of any technique used to extract the EEG activity in different frequency bands. Furthermore, recent evidence suggests that the presence of broadband activity of the EEG is required for a proper evaluation of complexity in the context of AD [27].

The reasons for the decrease of irregularity in the EEG of AD patients that are highlighted by FuzzyEn are not clear and might be a result of neuronal death, a consequence of neurotransmitter deficiency, and/or loss of connectivity of local neural networks as a result of nerve cell death [1]. These changes might be explained by the theory of AD being a disconnection syndrome [28]: the loss of connections between neurones in the cortex is a result from plaques and cell death [29], and this could lead to a much more regular EEG signal (recording of cortical brain activity).

Our pilot study has some limitations that should be mentioned. Although FuzzyEn is able to highlight subtle differences between EEG signals from AD patients and controls, the sample size used was small (11 AD patients and 11 control subjects). Therefore, the multiple comparisons might have resulted in an overestimation of the differences between the entropy of the EEG from AD patients and controls. Furthermore, the EEG changes that were detected by FuzzyEn might not be specific to AD. The detected increase of EEG regularity (or decrease of complexity) is also present in several physiological and pathological states, including, but not limited to, sleep [30], anaesthesia [31], the Creutzfeld-Jakob disease [32], vascular dementia [33], schizophrenia [34], or Parkinson’s disease [35]. Thus, future studies on FuzzyEn of the EEG in patients suffering from other dementias or mild cognitive impairment need to be completed to ascertain the possible usefulness of this signal processing method in the diagnosis of AD. 

Other potential future lines of research include the combination of FuzzyEn with MSE (used in [24] with SampEn as the entropy estimator) and the recently introduced refined composite MSE [36,37]. This could lead to further improvements in the characterisation of the EEG in AD. In fact, preliminary evidence suggests that refined multiscale FuzzyEn is able to detect differences due to AD in magnetoencephalograms [37]. Furthermore, a multivariate implementation of FuzzyEn could also be used in the analysis of the EEG in AD. This could potentially increase the discriminating power of the method, as shown with multivariate MSE with SampEn in [27]. However, it could also lead to the loss of the relevant spatial differences that are highlighted in this study (i.e., EEG changes in AD are not significant at all electrodes). Last, but not least, given that complementary information from EEG signals in AD can be highlighted by different methods, the combination of linear and non-linear signal processing algorithms could improve discrimination power. Among some of the entropy methods that could be tested, conditional entropy and corrected conditional entropy [38] and permutation entropy [39] show promise.

In summary, in spite of the aforementioned limitations, our findings with FuzzyEn suggest that this entropy estimator has potential to increase the insight into brain dysfunction in AD, as it detects subtle EEG differences between patients and controls with greater accuracy than SampEn or QSE. However, although our results generally support the notion of FuzzyEn outperforming these methods, as outlined in [17,19], one has to be very careful when comparing results, as that might be the case for certain combinations of input parameters, but not all.

## Figures and Tables

**Table 1:** Sensitivity, specificity, accuracy, and area under the ROC curve for FuzzyEn (*n* = 1) for all the electrodes and combinations of *m* and *r*, for which statistically significant differences between AD patients and control subjects were found. The threshold is the FuzzyEn value that maximises accuracy.

*m*	*r*	Electrode	Threshold	Sensitivity	Specificity	Accuracy	Area Under Curve
1	0.1	Fp1	1.1169	63.64	81.82	72.73	0.7934
		T6	1.3916	81.82	81.82	81.82	0.8182
		P3	1.1755	81.82	81.82	81.82	0.8595
		O2	1.2918	81.82	90.91	86.36	0.8595
1	0.15	Fp1	0.8015	63.64	81.82	72.73	0.7934
		T6	1.0217	81.82	81.82	81.82	0.8182
		P3	0.8516	81.82	90.91	86.36	0.8678
		O2	0.9393	81.82	90.91	86.36	0.8678
1	0.2	Fp1	0.6248	63.64	81.82	72.73	0.7934
		T6	0.8040	81.82	81.82	81.82	0.8182
		P3	0.6669	81.82	90.91	86.36	0.8678
		O2	0.7362	81.82	81.82	81.82	0.8554
1	0.25	Fp1	0.5105	63.64	81.82	72.73	0.7934
		T6	0.6662	81.82	81.82	81.82	0.8182
		P3	0.5473	81.82	90.91	86.36	0.8678
		O2	0.6054	81.82	81.82	81.82	0.8512
2	0.1	T6	0.8755	81.82	81.82	81.82	0.8182
		P3	0.7847	81.82	81.82	81.82	0.9091
		P4	0.7380	72.73	81.82	77.27	0.8099
		O1	0.8414	81.82	72.73	77.27	0.8264
		O2	0.8197	90.91	81.82	86.36	0.8512
2	0.15	T6	0.7127	81.82	81.82	81.82	0.8182
		P3	0.6295	81.82	81.82	81.82	0.8843
		P4	0.5885	63.64	90.91	77.27	0.8099
		O1	0.6879	81.82	72.73	77.27	0.8182
		O2	0.6617	90.91	81.82	86.36	0.8678
2	0.2	T6	0.6018	81.82	81.82	81.82	0.8264
		P3	0.5301	81.82	81.82	81.82	0.8926
		P4	0.4895	63.64	100	81.82	0.8182
		O1	0.5743	81.82	72.73	77.27	0.8099
		O2	0.5523	90.91	81.82	86.36	0.8595
2	0.25	T6	0.5206	81.82	81.82	81.82	0.8264
		P3	0.4564	81.82	81.82	81.82	0.9008
		P4	0.4182	63.64	100	81.82	0.8182
		O1	0.4926	81.82	72.73	77.27	0.8099
		O2	0.4727	90.91	81.82	86.36	0.8595

**Table 2:** Sensitivity, specificity, accuracy, and area under the ROC curve for FuzzyEn (*n* = 2) for all the electrodes and combinations of *m* and *r* for which statistically significant differences between AD patients and control subjects were found. The threshold is the FuzzyEn value that maximises accuracy.

*m*	*r*	Electrode	Threshold	Sensitivity	Specificity	Accuracy	Area Under Curve
1	0.1	P3	1.0782	81.82	81.82	81.82	0.8347
		O2	1.4320	72.73	81.82	77.27	0.8512
1	0.15	P3	0.8648	81.82	81.82	81.82	0.8264
		O2	1.1963	72.73	81.82	77.27	0.8430
1	0.2	P3	0.7279	81.82	81.82	81.82	0.8264
		O2	1.0326	72.73	81.82	77.27	0.8430
1	0.25	P3	0.6377	81.82	81.82	81.82	0.8264
2	0.15	P3	0.7627	81.82	81.82	81.82	0.8843
2	0.2	P3	0.7231	81.82	81.82	81.82	0.8843
		O2	0.7832	72.73	81.82	77.27	0.8264
2	0.25	P3	0.6875	81.82	81.82	81.82	0.8843
		O2	0.7493	72.73	81.82	77.27	0.8347

**Table 3:** Sensitivity, specificity, accuracy, and area under the receiver operating characteristic (ROC) curve for FuzzyEn (*n* = 3) for all the electrodes and combinations of *m* and *r* for which statistically significant differences between AD patients and control subjects were found. The threshold is the FuzzyEn value that maximises accuracy.

*m*	*r*	Electrode	Threshold	Sensitivity	Specificity	Accuracy	Area Under Curve
1	0.1	O2	1.4739	72.73	81.82	77.27	0.8430
1	0.15	O2	1.3153	72.73	81.82	77.27	0.8554
1	0.2	O2	1.2030	72.73	81.82	77.27	0.8678
1	0.25	O2	1.1147	72.73	81.82	77.27	0.8512
2	0.2	P3	0.7747	81.82	81.82	81.82	0.8306
2	0.25	P3	0.7571	81.82	81.82	81.82	0.8347

**Table 4:** Sensitivity, specificity, and accuracy for all the electrodes where significant differences between AD patients and control subjects were found with a selection of relevant non-linear methods previously used in the analysis of the same electroencephalogram (EEG) database.

Method	Electrode	ROC Classification Results		
		Sensitivity	Specificity	Accuracy
LZC (3 symbol conversion) [22]	T5	72.73	72.73	72.73
	P3	81.82	81.82	81.82
	P4	72.73	90.91	81.82
	O1	90.91	72.73	81.82
Slope of MSE (*m* = 1, *r* = 0.25, 12 scales) for large time scales [24]	F3	81.82	81.82	81.82
	F7	81.82	72.73	77.27
	Fp1	90.91	90.91	90.91
	Fp2	100	72.73	86.36
	T5	90.91	81.82	86.36
	T6	81.82	81.82	81.82
	P3	81.82	90.91	86.36
	P4	72.73	90.91	81.82
	O1	81.82	90.91	86.36
	O2	81.82	81.82	81.82
ApEn (*m* = 1, *r* = 0.25) [23]	P3	72.73	81.82	77.27
	P4	63.64	81.82	72.73
	O1	81.82	72.73	77.27
	O2	90.91	63.64	77.27
AMI rate of decrease [23]	T5	90.91	72.73	81.82
	T6	81.82	81.82	81.82
	P3	100	81.82	90.91
	P4	81.82	81.82	81.82
	O1	81.82	81.82	81.82
	O2	81.82	81.82	81.82
SampEn (*m* = 1, *r* = 0.25) [25]	P3	72.73	81.82	77.27
	P4	63.64	90.91	77.27
	O1	81.82	72.73	77.27
	O2	90.91	63.64	77.27
* QSE (*m* = 1 and *m* = 2, different values of *r*) [26]	P3	NR	NR	77.27
	P4	NR	NR	77.27
	O1	NR	NR	77.27
	O2	NR	NR	77.27

NR: not reported; * denotes the studies in which leave-one-subject-out cross-validation was used.

## Supplementary Materials

The following are available online at www.mdpi.com/1099-4300/20/1/21/s1, Table S1: FuzzyEn(*n* = 1, *m* = 1, *r* = 0.1) results, Table S2: FuzzyEn(*n* = 1, *m* = 1, *r* = 0.15) results, Table S3: FuzzyEn(*n* = 1, *m* = 1, *r* = 0.2) results, Table S4: FuzzyEn(*n* = 1, *m* = 1, *r* = 0.25) results, Table S5: FuzzyEn(*n* = 1, *m* = 2, *r* = 0.1) results, Table S6: FuzzyEn(*n* = 1, *m* = 2, *r* = 0.15) results, Table S7: FuzzyEn(*n* = 1, *m* = 2, *r* = 0.2) results, Table S8: FuzzyEn(*n* = 1, *m* = 2, *r* = 0.25) results, Table S9: FuzzyEn(*n* = 2, *m* = 1, *r* = 0.1) results, Table S10: FuzzyEn(*n* = 2, *m* = 1, *r* = 0.15) results, Table S11: FuzzyEn(*n* = 2, *m* = 1, *r* = 0.2) results, Table S12: FuzzyEn(*n* = 2, *m* = 1, *r* = 0.25) results, Table S13: FuzzyEn(*n* = 2, *m* = 2, *r* = 0.1) results, Table S14: FuzzyEn(*n* = 2, *m* = 2, *r* = 0.15) results, Table S15: FuzzyEn(*n* = 2, *m* = 2, *r* = 0.2) results, Table S16: FuzzyEn(*n* = 2, *m* = 2, *r* = 0.25) results, Table S17: FuzzyEn(*n* = 3, *m* = 1, *r* = 0.1) results, Table S18: FuzzyEn(*n* = 3, *m* = 1, *r* = 0.15) results, Table S19: FuzzyEn(*n* = 3, *m* = 1, *r* = 0.2) results, Table S20: FuzzyEn(*n* = 3, *m* = 1, *r* = 0.25) results, Table S21: FuzzyEn(*n* = 3, *m* = 2, *r* = 0.1) results, Table S22: FuzzyEn(*n* = 3, *m* = 2, *r* = 0.15) results, Table S23: FuzzyEn(*n* = 3, *m* = 2, *r* = 0.2) results, Table S24: FuzzyEn(*n* = 3, *m* = 2, *r* = 0.25) results.
