# Supplementary material for: Fuzzy Entropy Analysis of the Electroencephalogram in Patients with Alzheimer’s Disease: Is the Method Superior to Sample Entropy?
Source: Entropy (Basel). 2018 Jan 3;20(1):21. doi: 10.3390/e20010021 (PMC7512198; doi:10.3390/e20010021)
Supplement: Supplementary file 1 [file entropy-20-00021-s001.pdf]

entropy

Supplementary Material *Fuzzy Entropy Analysis of the Electroencephalogram in Patients with Alzheimer's Disease: Is the Method Superior to Sample Entropy? Samantha Simons, Pedro Espino and Daniel Abásolo*

Table S1. FuzzyEn(n=1,m=1,r=0.1) results. Significant differences (p<0.01) are highlighted in bold.

Electrode	Control subjects		AD patients		*p*-value
	Mean	SD	Mean	SD	
F3	1.3596	0.4729	1.0902	0.1013	0.0796
F4	1.2662	0.2878	1.2024	0.2862	0.6079
F7	1.3730	0.3878	1.3347	0.3318	0.8058
F8	1.3853	0.2915	1.3134	0.3560	0.6099
**Fp1**	**1.5287**	**0.4948**	**1.0296**	**0.2877**	**0.0090**
Fp2	1.4494	0.4282	1.0704	0.2658	0.0215
T3	1.7059	0.5225	1.5984	0.4042	0.5953
T4	1.6320	0.2727	1.7785	0.6767	0.5131
T5	1.5151	0.3280	1.2198	0.3260	0.0470
**T6**	**1.6719**	**0.4124**	**1.1845**	**0.3474**	**0.0071**
C3	1.7713	0.6258	1.2664	0.4854	0.0472
C4	1.5577	0.5128	1.3606	0.5267	0.3844
**P3**	**1.5730**	**0.4330**	**1.0417**	**0.3335**	**0.0043**
P4	1.4620	0.3209	1.0804	0.3679	0.0174
O1	1.6214	0.3960	1.2260	0.3326	0.0197
**O2**	**1.5597**	**0.2955**	**1.2001**	**0.2497**	**0.0059**

Table S2. FuzzyEn(n=1,m=1,r=0.15) results. Significant differences (p<0.01) are highlighted in bold.

Electrode	Control subjects		AD patients		*p*-value
	Mean	SD	Mean	SD	
F3	0.9936	0.3579	0.7879	0.0791	0.0775
F4	0.9207	0.2134	0.8766	0.2153	0.6347
F7	1.0105	0.3075	0.9781	0.2590	0.7921
F8	1.0169	0.2207	0.9603	0.2728	0.5984
**Fp1**	**1.1087**	**0.3548**	**0.7427**	**0.2175**	**0.0085**
Fp2	1.0515	0.3244	0.7707	0.1994	0.0238
T3	1.2803	0.4202	1.1956	0.3257	0.6030
T4	1.2168	0.2241	1.3419	0.5598	0.4991
T5	1.1260	0.2655	0.8869	0.2514	0.0423
**T6**	**1.2354**	**0.3097**	**0.8651**	**0.2683**	**0.0071**
C3	1.3013	0.4738	0.9276	0.3747	0.0535
C4	1.1507	0.3925	1.0038	0.4157	0.4044
**P3**	**1.1562**	**0.3187**	**0.7539**	**0.2554**	**0.0039**
P4	1.0778	0.2437	0.7831	0.2767	0.0153
O1	1.2141	0.3185	0.8955	0.2630	0.0187
**O2**	**1.1640**	**0.2416**	**0.8754**	**0.2039**	**0.0066**

Table S3. FuzzyEn(n=1,m=1,r=0.2) results. Significant differences (p<0.01) are highlighted in bold.

Electrode	Control subjects		AD patients		p-value
	Mean	SD	Mean	SD	
F3	0.7810	0.2861	0.6160	0.0646	0.0769
F4	0.7223	0.1700	0.6877	0.1715	0.6399
F7	0.7974	0.2509	0.7705	0.2106	0.7878
F8	0.8018	0.1774	0.7556	0.2198	0.5933
**Fp1**	**0.8678**	**0.2756**	**0.5802**	**0.1739**	**0.0083**
Fp2	0.8237	0.2603	0.6014	0.1588	0.0253
T3	1.0219	0.3474	0.9533	0.2710	0.6116
T4	0.9677	0.1876	1.0748	0.4706	0.4911
T5	0.8936	0.2197	0.6956	0.2031	0.0402
**T6**	**0.9774**	**0.2477**	**0.6803**	**0.2174**	**0.0072**
C3	1.0247	0.3761	0.7305	0.3032	0.0570
C4	0.9094	0.3142	0.7935	0.3399	0.4165
**P3**	**0.9119**	**0.2506**	**0.5898**	**0.2054**	**0.0036**
P4	0.8517	0.1955	0.6132	0.2206	0.0143
O1	0.9675	0.2631	0.7041	0.2154	0.0183
**O2**	**0.9259**	**0.2012**	**0.6878**	**0.1696**	**0.0071**

Table S4. FuzzyEn(n=1,m=1,r=0.25) results. Significant differences (p<0.01) are highlighted in bold.

Electrode	Control subjects		AD patients		p-value
	Mean	SD	Mean	SD	
F3	0.6428	0.2379	0.5055	0.0545	0.0768
F4	0.5940	0.1413	0.5653	0.1422	0.6404
F7	0.6578	0.2106	0.6351	0.1769	0.7870
F8	0.6613	0.1482	0.6224	0.1837	0.5906
**Fp1**	**0.7124**	**0.2252**	**0.4757**	**0.1446**	**0.0082**
Fp2	0.6766	0.2171	0.4928	0.1317	0.0262
T3	0.8492	0.2949	0.7921	0.2313	0.6187
T4	0.8025	0.1605	0.8954	0.4036	0.4862
T5	0.7400	0.1865	0.5719	0.1699	0.0389
**T6**	**0.8078**	**0.2062**	**0.5602**	**0.1823**	**0.0073**
C3	0.8441	0.3107	0.6021	0.2540	0.0592
C4	0.7508	0.2610	0.6555	0.2866	0.4243
**P3**	**0.7523**	**0.2062**	**0.4840**	**0.1713**	**0.0034**
P4	0.7034	0.1630	0.5037	0.1830	0.0137
O1	0.8032	0.2231	0.5796	0.1817	0.0180
**O2**	**0.7678**	**0.1715**	**0.5660**	**0.1445**	**0.0073**

Table S5. FuzzyEn(n=1,m=2,r=0.1) results. Significant differences (p<0.01) are highlighted in bold.

Electrode	Control subjects		AD patients		*p*-value
	Mean	SD	Mean	SD	
F3	0.8233	0.1714	0.7405	0.0612	0.1472
F4	0.8086	0.1345	0.7803	0.1233	0.6131
F7	0.8542	0.1633	0.8376	0.1262	0.7920
F8	0.8452	0.1191	0.8247	0.1390	0.7149
Fp1	0.8896	0.1837	0.6943	0.1589	0.0148
Fp2	0.8769	0.1744	0.7240	0.1401	0.0346
T3	0.9588	0.1830	0.9331	0.1492	0.7220
T4	0.9408	0.1243	0.9553	0.2227	0.8520
T5	0.9108	0.1172	0.7887	0.1310	0.0321
**T6**	**0.9626**	**0.1732**	**0.7620**	**0.1550**	**0.0096**
C3	0.9799	0.1718	0.7898	0.1789	0.0194
C4	0.8885	0.1305	0.8147	0.1690	0.2652
**P3**	**0.9126**	**0.1274**	**0.6940**	**0.1158**	**0.0004**
**P4**	**0.8696**	**0.1068**	**0.7152**	**0.1359**	**0.0077**
**O1**	**0.9254**	**0.1345**	**0.7692**	**0.1119**	**0.0077**
**O2**	**0.9150**	**0.1160**	**0.7573**	**0.0970**	**0.0025**

Table S6. FuzzyEn(n=1,m=2,r=0.15) results. Significant differences (p<0.01) are highlighted in bold.

Electrode	Control subjects		AD patients		*p*-value
	Mean	SD	Mean	SD	
F3	0.6729	0.1608	0.5872	0.0547	0.1098
F4	0.6505	0.1177	0.6285	0.1134	0.6604
F7	0.6909	0.1471	0.6762	0.1167	0.7980
F8	0.6869	0.1098	0.6628	0.1248	0.6358
Fp1	0.7254	0.1634	0.5491	0.1408	0.0135
Fp2	0.7070	0.1593	0.5726	0.1277	0.0411
T3	0.7989	0.1751	0.7653	0.1372	0.6222
T4	0.7758	0.1104	0.7873	0.2049	0.8718
T5	0.7545	0.1135	0.6329	0.1227	0.0255
**T6**	**0.7995**	**0.1525**	**0.6161**	**0.1420**	**0.0076**
C3	0.8124	0.1636	0.6396	0.1649	0.0228
C4	0.7365	0.1335	0.6673	0.1662	0.2944
**P3**	**0.7550**	**0.1232**	**0.5520**	**0.1109**	**0.0006**
**P4**	**0.7204**	**0.1001**	**0.5720**	**0.1301**	**0.0071**
**O1**	**0.7757**	**0.1332**	**0.6216**	**0.1079**	**0.0074**
**O2**	**0.7578**	**0.1074**	**0.6108**	**0.0911**	**0.0024**

Table S7. FuzzyEn(n=1,m=2,r=0.2) results. Significant differences (p<0.01) are highlighted in bold.

Electrode	Control subjects		AD patients		*p*-value
	Mean	SD	Mean	SD	
F3	0.5658	0.1459	0.4853	0.0491	0.0983
F4	0.5422	0.1026	0.5264	0.1015	0.7211
F7	0.5807	0.1356	0.5661	0.1073	0.7833
F8	0.5777	0.0994	0.5534	0.1123	0.5972
Fp1	0.6083	0.1429	0.4529	0.1236	0.0129
Fp2	0.5897	0.1436	0.4716	0.1118	0.0438
T3	0.6867	0.1673	0.6505	0.1282	0.5746
T4	0.6616	0.1029	0.6753	0.1970	0.8399
T5	0.6430	0.1092	0.5271	0.1120	0.0232
**T6**	**0.6811**	**0.1347**	**0.5123**	**0.1288**	**0.0070**
C3	0.6915	0.1572	0.5369	0.1518	0.0294
C4	0.6270	0.1285	0.5650	0.1595	0.3270
**P3**	**0.6409**	**0.1127**	**0.4574**	**0.1023**	**0.0007**
**P4**	**0.6125**	**0.0916**	**0.4755**	**0.1188**	**0.0066**
**O1**	**0.6677**	**0.1589**	**0.5204**	**0.1019**	**0.0075**
**O2**	**0.6473**	**0.1026**	**0.5108**	**0.0861**	**0.0030**

Table S8. FuzzyEn(n=1,m=2,r=0.25) results. Significant differences (p<0.01) are highlighted in bold.

Electrode	Control subjects		AD patients		*p*-value
	Mean	SD	Mean	SD	
F3	0.4863	0.1313	0.4128	0.0443	0.0939
F4	0.4638	0.0907	0.4517	0.0908	0.7566
F7	0.5001	0.1246	0.4860	0.0979	0.7703
F8	0.4974	0.0898	0.4742	0.1010	0.5765
Fp1	0.5213	0.1249	0.3847	0.1090	0.0128
Fp2	0.6010	0.1571	0.5652	0.1192	0.0452
T3	0.5757	0.0957	0.5913	0.1876	0.5537
T4	0.5757	0.0957	0.5913	0.1876	0.8076
T5	0.5585	0.1025	0.4506	0.1015	0.0222
**T6**	**0.5911**	**0.1198**	**0.4393**	**0.1165**	**0.0069**
C3	0.5991	0.1458	0.4617	0.1387	0.0349
C4	0.5438	0.1196	0.4887	0.1493	0.3514
**P3**	**0.5546**	**0.1013**	**0.3898**	**0.0930**	**0.0007**
**P4**	**0.5309**	**0.0831**	**0.4060**	**0.1072**	**0.0063**
**O1**	**0.5842**	**0.1214**	**0.4466**	**0.0947**	**0.0077**
**O2**	**0.5636**	**0.0966**	**0.4381**	**0.0805**	**0.0035**

Table S9. FuzzyEn(n=2,m=1,r=0.1) results. Significant differences (p<0.01) are highlighted in bold.

Electrode	Control subjects		AD patients		*p*-value
	Mean	SD	Mean	SD	
F3	1.3286	0.5713	1.1774	0.2380	0.8182
F4	1.2123	0.3400	1.2213	0.2897	0.9738
F7	1.5304	0.5362	1.6788	0.4409	0.3754
F8	1.5417	0.3989	1.6295	0.5307	0.7180
Fp1	1.8758	0.8766	1.2847	0.4084	0.1077
Fp2	1.7550	0.6921	1.3241	0.4081	0.1228
T3	2.0140	0.7546	2.0202	0.5317	0.6695
T4	1.8817	0.4566	2.3943	1.0586	0.3410
T5	1.6875	0.5488	1.4238	0.3087	0.2505
T6	2.1967	1.0107	1.3660	0.4421	0.0197
C3	1.7349	0.8115	1.2220	0.6179	0.0818
C4	1.4060	0.5794	1.3084	0.5845	0.6224
**P3**	**1.6226**	**0.5988**	**1.0352**	**0.3027**	**0.0078**
P4	1.4989	0.4586	1.0792	0.4188	0.0138
O1	1.7959	0.4461	1.3924	0.3374	0.0386
**O2**	**1.7561**	**0.2886**	**1.3667**	**0.2266**	**0.0053**

Table S10. FuzzyEn(n=2,m=1,r=0.15) results. Significant differences (p<0.01) are highlighted in bold.

Electrode	Control subjects		AD patients		*p*-value
	Mean	SD	Mean	SD	
F3	1.0991	0.5110	0.9669	0.2162	0.8696
F4	0.9860	0.2869	1.0050	0.2584	0.9215
F7	1.2891	0.4947	1.4397	0.4199	0.3410
F8	1.2918	0.3386	1.3895	0.4780	0.6695
Fp1	1.5622	0.7379	1.0739	0.3854	0.0818
Fp2	1.4584	0.5913	1.1009	0.3768	0.1228
T3	1.7381	0.6766	1.7612	0.4851	0.7180
T4	1.6144	0.4212	2.0714	0.9149	0.2244
T5	1.4460	0.5085	1.1900	0.2761	0.2244
T6	1.8357	0.7881	1.1443	0.3945	0.0197
C3	1.4291	0.7072	1.0064	0.5480	0.0818
C4	1.1624	0.5182	1.0841	0.5309	0.6695
**P3**	**1.3490**	**0.5190**	**0.8386**	**0.2689**	**0.0095**
P4	1.2464	0.3828	0.8763	0.3659	0.0138
O1	1.5564	0.4253	0.1708	0.3101	0.0386
**O2**	**1.5189**	**0.2790**	**1.1465**	**0.2176**	**0.0064**

Table S11. FuzzyEn(n=2,m=1,r=0.2) results. Significant differences (p<0.01) are highlighted in bold.

Electrode	Control subjects		AD patients		*p*-value
	Mean	SD	Mean	SD	
F3	0.9443	0.4629	0.8264	0.1983	0.8182
F4	0.8359	0.2507	0.8605	0.2339	0.9215
F7	1.1278	0.4666	1.2734	0.4035	0.3410
F8	1.1237	0.3023	1.2234	0.4398	0.5767
Fp1	1.3560	0.6527	0.9319	0.3637	0.0818
Fp2	1.2623	0.5312	0.9505	0.3505	0.1228
T3	1.5510	0.6311	1.5833	0.4549	0.7180
T4	1.4315	0.4010	1.8604	0.8413	0.2505
T5	1.2803	0.4852	1.0303	0.2505	0.2004
T6	1.6052	0.6748	0.9945	0.3625	0.0197
C3	1.2296	0.6430	0.8636	0.4982	0.1077
C4	0.9984	0.4713	0.9348	0.4921	0.6695
**P3**	**1.1678**	**0.4678**	**0.7094**	**0.2421**	**0.0095**
P4	1.0786	0.3394	0.7435	0.3279	0.0165
O1	1.3896	0.4078	1.0183	0.2876	0.0386
**O2**	**1.3527**	**0.2727**	**0.9954**	**0.2079**	**0.0064**

Table S12. FuzzyEn(n=2,m=1,r=0.25) results. Significant differences (p<0.01) are highlighted in bold.

Electrode	Control subjects		AD patients		*p*-value
	Mean	SD	Mean	SD	
F3	0.8306	0.4231	0.7240	0.1831	0.8696
F4	0.7276	0.2241	0.7555	0.2140	0.8182
F7	1.0088	0.4441	1.1475	0.3890	0.3410
F8	0.9992	0.2761	1.0982	0.4094	0.5327
Fp1	1.2048	0.5899	0.8273	0.3438	0.0818
Fp2	1.1187	0.4870	0.8403	0.3281	0.1228
T3	1.4105	0.5978	1.4484	0.4319	0.7676
T4	1.2943	0.3857	1.7046	0.7936	0.2505
T5	1.1558	0.4681	0.9118	0.2295	0.1396
T6	1.4386	0.6034	0.8839	0.3376	0.0278
C3	1.0855	0.5945	0.7597	0.4587	0.0940
C4	0.8779	0.4328	0.8259	0.4608	0.7180
**P3**	**1.0350**	**0.4283**	**0.6166**	**0.2203**	**0.0095**
P4	0.9557	0.3086	0.6481	0.2980	0.0138
O1	1.2626	0.3920	0.9045	0.2684	0.0328
O2	1.2261	0.2669	0.8830	0.1980	0.0115

Table S13. FuzzyEn(n=2,m=2,r=0.1) results. Significant differences (p<0.01) are highlighted in bold.

Electrode	Control subjects		AD patients		*p*-value
	Mean	SD	Mean	SD	
F3	0.9145	0.1808	0.9118	0.1485	0.8696
F4	0.8827	0.1255	0.8926	0.0829	0.8182
F7	1.1658	0.4328	1.1157	0.2937	0.7180
F8	1.0458	0.2281	1.0629	0.2479	0.7676
Fp1	1.2069	0.5184	0.9221	0.1473	0.2244
Fp2	1.1822	0.4702	0.9907	0.2624	0.2786
T3	1.2637	0.5150	1.2921	0.2857	0.3754
T4	1.2066	0.3894	1.6018	0.7636	0.3410
T5	1.1425	0.4386	0.9769	0.1317	0.2505
T6	1.4480	0.9440	0.9692	0.2340	0.0452
C3	1.3398	0.9259	0.8854	0.2368	0.0197
C4	0.9369	0.1680	0.9201	0.2383	0.6224
**P3**	**1.1001**	**0.3404**	**0.8119**	**0.0733**	**0.0023**
P4	0.9820	0.2022	0.8453	0.1410	0.0328
O1	1.0817	0.1922	0.9127	0.0947	0.0452
O2	1.0902	0.1893	0.9155	0.1120	0.0278

Table S14. FuzzyEn(n=2,m=2,r=0.15) results. Significant differences (p<0.01) are highlighted in bold.

Electrode	Control subjects		AD patients		*p*-value
	Mean	SD	Mean	SD	
F3	0.8395	0.1687	0.8277	0.1161	0.8696
F4	0.8206	0.1386	0.8236	0.0863	0.7180
F7	1.0105	0.3040	1.0027	0.2131	0.6224
F8	0.9347	0.1643	0.9594	0.1997	0.8696
Fp1	1.0493	0.3667	0.8387	0.1374	0.2244
Fp2	1.0309	0.3214	0.8903	0.1956	0.2505
T3	1.1113	0.3607	1.1361	0.2172	0.4502
T4	1.0714	0.2724	1.3341	0.5450	0.4502
T5	1.0211	0.3186	0.8928	0.1062	0.1396
T6	1.2304	0.6401	0.8759	0.1883	0.0328
C3	1.1376	0.6044	0.8017	0.1986	0.0138
C4	0.8561	0.1474	0.8378	0.1932	0.5767
**P3**	**0.9801**	**0.2353**	**0.7471**	**0.0777**	**0.0023**
P4	0.8892	0.1455	0.7717	0.1289	0.0278
O1	0.9773	0.1558	0.8415	0.0837	0.0452
O2	0.9880	0.1447	0.8369	0.0855	0.0165

Table S15. FuzzyEn(n=2,m=2,r=0.2) results. Significant differences (p<0.01) are highlighted in bold.

Electrode	Control subjects		AD patients		*p*-value
	Mean	SD	Mean	SD	
F3	0.7904	0.1714	0.7724	0.1045	0.8182
F4	0.7744	0.1464	0.7751	0.0924	0.8182
F7	0.9228	0.2452	0.9371	0.1727	0.6224
F8	0.8704	0.1387	0.8991	0.1799	0.7676
Fp1	0.9659	0.3033	0.7841	0.1373	0.2244
Fp2	0.9498	0.2565	0.8280	0.1702	0.2505
T3	1.0248	0.2866	1.0466	0.1824	0.5767
T4	0.9932	0.2127	1.1884	0.4322	0.5327
T5	0.9509	0.2573	0.8410	0.0990	0.2244
T6	1.1158	0.4896	0.8179	0.1682	0.0328
C3	1.0275	0.4472	0.7477	0.1876	0.0165
C4	0.8053	0.1443	0.7832	0.1754	0.5327
**P3**	**0.9114**	**0.1925**	**0.6997**	**0.0843**	**0.0023**
P4	0.8344	0.1240	0.7203	0.1283	0.0197
O1	0.9165	0.1372	0.7968	0.0821	0.0386
**O2**	**0.9278**	**0.1210**	**0.7924**	**0.0749**	**0.0095**

Table S16. FuzzyEn(n=2,m=2,r=0.25) results. Significant differences (p<0.01) are highlighted in bold.

Electrode	Control subjects		AD patients		*p*-value
	Mean	SD	Mean	SD	
F3	0.7519	0.1766	0.7296	0.1002	0.8182
F4	0.7351	0.1488	0.7356	0.0976	0.8182
F7	0.8631	0.2144	0.8912	0.1504	0.4905
F8	0.8254	0.1276	0.8569	0.1707	0.7180
Fp1	0.9115	0.2747	0.7428	0.1403	0.1783
Fp2	0.8959	0.2268	0.7814	0.1593	0.2244
T3	0.9669	0.2455	0.9868	0.1624	0.5327
T4	0.9394	0.1783	1.0950	0.3640	0.5767
T5	0.9025	0.2215	0.8024	0.0976	0.2244
T6	1.0431	0.4020	0.7753	0.1586	0.0235
C3	0.9531	0.3585	0.7065	0.1855	0.0278
C4	0.7665	0.1465	0.7411	0.1685	0.6224
**P3**	**0.8632**	**0.1749**	**0.6606**	**0.0901**	**0.0023**
P4	0.7948	0.1164	0.6793	0.1309	0.0165
O1	0.8748	0.1280	0.7629	0.0840	0.0386
**O2**	**0.8858**	**0.1077**	**0.7570**	**0.0711**	**0.0078**

Table S17. FuzzyEn(n=3,m=1,r=0.1) results. Significant differences (p<0.01) are highlighted in bold.

Electrode	Control subjects		AD patients		*p*-value
	Mean	SD	Mean	SD	
F3	1.2795	0.5952	1.1876	0.3097	0.8696
F4	1.1558	0.3534	1.1981	0.2958	0.7180
F7	1.6039	0.6476	1.7706	0.4828	0.2786
F8	1.6591	0.6648	1.7308	0.6521	0.6695
Fp1	2.2795	1.7058	1.3682	0.4450	0.2244
Fp2	2.0961	1.4025	1.4049	0.4612	0.1396
T3	2.3054	1.3268	2.2791	0.7874	0.7180
T4	2.0778	0.8977	3.5472	3.3659	0.3088
T5	1.8327	1.0390	1.4781	0.3261	0.4502
T6	3.7203	4.4851	1.4424	0.5738	0.0278
C3	1.7926	1.0970	1.1908	0.7139	0.1396
C4	1.3108	0.5924	1.2631	0.6337	0.7180
P3	1.6931	0.8110	1.0122	0.2943	0.0197
P4	1.5869	0.8488	1.0635	0.4549	0.0452
O1	1.8168	0.4765	1.4167	0.3407	0.0452
**O2**	**1.7840**	**0.3225**	**1.3947**	**0.2360**	**0.0064**

Table S18. FuzzyEn(n=3,m=1,r=0.15) results. Significant differences (p<0.01) are highlighted in bold.

Electrode	Control subjects		AD patients		*p*-value
	Mean	SD	Mean	SD	
F3	1.1201	0.5553	1.0385	0.2912	0.8696
F4	1.0004	0.3195	1.0491	0.2805	0.6695
F7	1.4066	0.5804	1.6041	0.4615	0.2244
F8	1.4397	0.5075	1.5529	0.5864	0.5767
Fp1	1.9183	1.2542	0.2165	0.4342	0.2244
Fp2	1.7667	1.0223	1.2466	0.4424	0.1396
T3	2.0189	1.0489	2.0348	0.6574	0.6695
T4	1.8304	0.7037	2.9423	2.4098	0.3088
T5	1.6158	0.8476	1.3122	0.2966	0.3754
T6	2.9388	3.0736	1.2715	0.4905	0.0278
C3	1.5203	0.8795	1.0323	0.6372	0.1396
C4	1.1403	0.5479	1.1022	0.5821	0.8182
P3	1.4532	0.6653	0.8738	0.2764	0.0235
P4	1.3617	0.6350	0.9160	0.4076	0.0527
O1	1.6467	0.4579	1.2669	0.3250	0.0452
**O2**	**1.6200**	**0.3068**	**1.2430**	**0.2292**	**0.0053**

Table S19. FuzzyEn(n=3,m=1,r=0.2) results. Significant differences (p<0.01) are highlighted in bold.

Electrode	Control subjects		AD patients		*p*-value
	Mean	SD	Mean	SD	
F3	1.0105	0.5254	0.9365	0.2790	0.8696
F4	0.8930	0.2948	0.9455	0.2683	0.6695
F7	1.2785	0.5463	1.4882	0.4489	0.2244
F8	1.2994	0.4333	1.4323	0.5499	0.5327
Fp1	1.7041	1.0407	1.1118	0.4255	0.2244
Fp2	1.5710	0.8456	1.1369	0.4291	0.1580
T3	1.8423	0.9146	1.8804	0.5945	0.6695
T4	1.6725	0.6146	2.6075	1.9381	0.3410
T5	1.4766	0.7531	1.1973	0.2783	0.3410
T6	2.5142	2.3735	1.1568	0.4478	0.0328
C3	1.3501	0.7792	0.9270	0.5912	0.1580
C4	1.0242	0.5167	0.9931	0.5502	0.8182
P3	1.3016	0.5947	0.7789	0.2620	0.0235
P4	1.2189	0.5323	0.8166	0.3782	0.0613
O1	1.5303	0.4463	1.1608	0.3125	0.0527
**O2**	**1.5052**	**0.2998**	**1.1367**	**0.2250**	**0.0035**

Table S20. FuzzyEn(n=3,m=1,r=0.25) results. Significant differences (p<0.01) are highlighted in bold.

Electrode	Control subjects		AD patients		*p*-value
	Mean	SD	Mean	SD	
F3	0.9276	0.5006	0.8599	0.2694	0.8696
F4	0.8121	0.2754	0.8669	0.2580	0.5327
F7	1.1848	0.5247	1.3995	0.4400	0.1783
F8	1.1975	0.3901	1.3413	0.5247	0.4905
Fp1	1.5558	0.9175	1.0324	0.4177	0.2244
Fp2	1.4354	0.7457	1.0535	0.4180	0.1580
T3	1.7165	0.8356	1.7686	0.5567	0.6695
T4	1.5575	0.5648	2.3873	1.6587	0.3088
T5	1.3751	0.6966	1.1097	0.2647	0.2786
T6	2.2395	1.9568	1.0711	0.4208	0.0328
C3	1.2285	0.7202	0.8492	0.5582	0.1580
C4	0.9370	0.4919	0.9116	0.5270	0.8696
P3	1.1925	0.5513	0.7078	0.2499	0.0235
P4	1.1157	0.4719	0.7427	0.3566	0.0613
O1	1.4416	0.4375	1.0790	0.3020	0.0613
**O2**	**1.4169**	**0.2959**	**1.0554**	**0.2217**	**0.0053**

Table S21. FuzzyEn(n=3,m=2,r=0.1) results.

Electrode	Control subjects		AD patients		p-value
	Mean	SD	Mean	SD	
F3	0.9753	0.3331	1.0884	0.6098	0.6224
F4	0.8696	0.1222	0.8981	0.0859	0.5767
F7	1.9259	1.6976	1.4645	0.9514	0.9738
F8	1.5020	1.1510	1.3010	0.7338	0.8696
Fp1	2.4349	3.1358	0.9939	0.1776	0.4118
Fp2	2.3398	2.9058	1.5332	1.8197	0.3754
T3	2.2397	3.0194	2.1321	1.6342	0.2004
T4	1.9524	2.2998	5.0861	6.1328	0.2004
T5	2.0593	3.2465	1.1593	0.6239	0.4118
T6	5.0072	9.6638	1.1840	0.7904	0.0940
C3	2.7720	4.6907	0.9625	0.4422	0.0527
C4	0.9799	0.3254	1.0061	0.5250	0.7676
P3	1.6640	1.5469	0.8184	0.0753	0.0165
P4	1.2381	0.8679	0.8977	0.2522	0.1077
O1	1.1726	0.2993	0.9352	0.1046	0.0452
O2	1.1861	0.3503	1.0092	0.3432	0.0527

Table S22. FuzzyEn(n=3,m=2,r=0.15) results.

Electrode	Control subjects		AD patients		p-value
	Mean	SD	Mean	SD	
F3	0.8971	0.2471	0.9694	0.4126	0.7676
F4	0.8309	0.1270	0.8495	0.0805	0.6695
F7	1.5421	1.1410	1.2631	0.6605	0.8182
F8	1.2589	0.7668	1.1475	0.5129	0.7180
Fp1	1.8772	2.0846	0.9268	0.1535	0.3410
Fp2	1.8166	1.9253	1.2782	1.2109	0.2786
T3	1.7946	2.0251	1.7405	1.1055	0.2004
T4	1.5997	1.5521	3.7076	4.1414	0.2004
T5	1.6593	2.2063	1.0350	0.4208	0.4118
T6	3.6236	6.4546	1.0482	0.5434	0.0613
C3	2.0999	3.1130	0.8718	0.3255	0.0386
C4	0.8977	0.2378	0.9178	0.3790	0.7676
P3	1.3704	1.0281	0.7786	0.0714	0.0138
P4	1.0747	0.5763	0.8318	0.1927	0.0818
O1	1.0685	0.2420	0.8778	0.0918	0.0386
O2	1.0822	0.2640	0.9245	0.2356	0.0527

Table S23. FuzzyEn(n=3,m=2,r=0.2) results. Significant differences (p<0.01) are highlighted in bold.

Electrode	Control subjects		AD patients		*p*-value
	Mean	SD	Mean	SD	
F3	0.8522	0.2122	0.9025	0.3158	0.7676
F4	0.8046	0.1341	0.8185	0.0803	0.5767
F7	1.3424	0.8625	1.1553	0.5138	0.8696
F8	1.1309	0.5748	1.0623	0.4035	0.7180
Fp1	1.5943	1.5606	0.8845	0.1411	0.3754
Fp2	1.5488	1.4350	1.1442	0.9083	0.2786
T3	1.5631	1.5281	1.5331	0.8404	0.2244
T4	1.4145	1.1752	3.0055	3.1414	0.2505
T5	1.4509	1.6829	0.9674	0.3202	0.3088
T6	2.9258	4.8485	0.9725	0.4220	0.0452
C3	1.7606	2.3232	0.8185	0.2711	0.0386
C4	0.8509	0.1995	0.8657	0.3073	0.8182
**P3**	**1.2184**	**0.7690**	**0.7511**	**0.0720**	**0.0095**
P4	0.9877	0.4326	0.7914	0.1662	0.0818
O1	1.0068	0.2099	0.8434	0.0845	0.0527
O2	1.0214	0.2199	0.8763	0.1818	0.0452

Table S24. FuzzyEn(n=3,m=2,r=0.25) results. Significant differences (p<0.01) are highlighted in bold.

Electrode	Control subjects		AD patients		*p*-value
	Mean	SD	Mean	SD	
F3	0.8214	0.1967	0.8578	0.2592	0.8182
F4	0.7838	0.1403	0.7954	0.0820	0.5767
F7	1.2182	0.6957	1.0867	0.4252	0.6695
F8	1.0505	0.4602	1.0066	0.3391	0.7180
Fp1	1.4224	1.2472	0.8538	0.1342	0.3754
Fp2	1.3847	1.1412	1.0600	0.7281	0.3088
T3	1.4193	1.2300	1.4022	0.6810	0.2505
T4	1.2986	0.9476	2.5775	2.5389	0.3754
T5	1.3213	1.3671	0.9238	0.2607	0.3754
T6	2.5040	3.8842	0.9227	0.3508	0.0328
C3	1.5543	1.8485	0.7818	0.2420	0.0613
C4	0.8188	0.1801	0.8294	0.2660	0.8182
**P3**	**1.1238**	**0.6146**	**0.7294**	**0.0743**	**0.0078**
P4	0.9321	0.3479	0.7622	0.1529	0.0613
O1	0.9649	0.1889	0.8197	0.0805	0.0818
O2	0.9804	0.1925	0.8439	0.1494	0.0386

© 2018 by the authors. Submitted for possible open access publication under the terms and conditions of the Creative Commons Attribution (CC-BY) license (http://creativecommons.org/licenses/by/4.0/).
